# Ecophysiological Traits May Explain the Abundance of Climbing Plant Species across the Light Gradient in a Temperate Rainforest

**DOI:** 10.1371/journal.pone.0038831

**Published:** 2012-06-07

**Authors:** Ernesto Gianoli, Alfredo Saldaña, Mylthon Jiménez-Castillo

**Affiliations:** 1 Departamento de Biología, Universidad de La Serena, La Serena, Chile; 2 Departamento de Botánica, Universidad de Concepción, Concepción, Chile; 3 Center for Advanced Studies in Ecology and Biodiversity, Pontificia Universidad Católica de Chile, Santiago, Chile; 4 Instituto de Ciencias Ambientales y Evolutivas, Universidad Austral de Chile, Valdivia, Chile; Jyväskylä University, Finland

## Abstract

Climbing plants are a key component of rainforests, but mechanistic approaches to their distribution and abundance are scarce. In a southern temperate rainforest, we addressed whether the dominance of climbing plants across light environments is associated with the expression of ecophysiological traits. In mature forest and canopy gaps, we measured leaf size, specific leaf area, photosynthetic rate, and dark respiration in six of the most abundant woody vines. Mean values of traits and their phenotypic change (%) between mature forest and canopy gaps were predictor variables. Leaf size and specific leaf area were not significantly associated with climbing plant dominance. Variation in gas-exchange traits between mature forest and canopy gaps explained, at least partly, the dominance of climbers in this forest. A greater increase in photosynthetic rate and a lower increase in dark respiration rate when canopy openings occur were related to the success of climbing plant species. Dominant climbers showed a strategy of maximizing exploitation of resource availability but minimizing metabolic costs. Results may reflect phenotypic plasticity or genetic differentiation in ecophysiological traits between light environments. It is suggested that the dominant climbers in this temperate rainforest would be able to cope with forest clearings due to human activities.

## Introduction

Rainforests are heterogeneous ecosystems in terms of light availability [1], [2] and show as a distinctive feature a considerable abundance of climbing plants [3]–[9]. This suggests that climbing plants, as a group, should be able to thrive under a range of light intensities. The relationship between light availability and climbing plant density has been evaluated in several rainforests worldwide (references in [4], [9]). Overall, it seems that climbing plants show similar abundances across forest light gradients. Certainly, not all climbing plant species exhibit an even density throughout canopy gaps, secondary forest and mature forest; some species behave as light-demanding pioneers and some proliferate in the shaded understory of mature forests [3], [4], [9], [10].

Functional explanations for the distribution of tree species with regard to light availability are widely available in the literature (reviewed in [11]), while there is little research on mechanistic explanations for distribution patterns of climbing plants across the light gradient. There is evidence of a relationship between the species' climbing mechanism and both photosynthetic acclimation and abundance in contrasting light environments [12]–[14]. A field study recently reported that the dominance of climbing plant species in a shaded forest understory was positively related to light-interception efficiency and inversely related to potential carbon gain [15]. To our knowledge, no research has attempted to link the distribution and abundance of climbing plant species in contrasting light environments with the expression of ecophysiological traits related to light exploitation. This research question is of interest from a basic perspective, i.e., Do climbing plants show strategies of adaptation to light heterogeneity similar to those exhibited by trees?, considering that vines may account for 40% of species diversity in tropical forests [4], are present in more than 130 plant families [16], and the climbing habit is associated with increased diversification in plant lineages [17]. This question is also of interest to estimate the response of a key component of rainforest communities to changes in climate or land-use [8], [18], [19], considering evidence that woody climbers are increasing in dominance, relative to trees, in several forests worldwide [20]–[23]. Here, we report results of a field study in a temperate rainforest showing a significant association between the dominance of climbing plant species across light environments and changes in ecophysiological traits between forest understory and canopy gaps.

## Materials and Methods

The study was carried out in a temperate evergreen rainforest in Anticura, Southern Chile (40°39′S, 72°11′W; site and community details described in [9]). A research permit to conduct field work in Anticura, which is located within Puyehue National Park, was obtained from CONAF (National Forestry Corporation, Permit N° 17/2010). Annual precipitation in the study site is 2800 mm and mean annual temperature is 9.8 °C, with potential evapotranspiration only 1/8th of precipitation rates [24]. The abundance of climbing plant species was determined in 45 plots of 25 m^2^, 15 plots in each of three distinct light environments within the forest [9]. We considered the following light environments, where light availability was quantified as Global Site Factor (GSF, the proportion of total radiation reaching a plot) using hemispherical photographs: closed canopy old-growth forest (mean GSF, 0.062), secondary forest stands (mean GSF, 0.098) and treefall canopy gaps (mean GSF, 0.187) [9]. Plots were located within an area of ca. 30 ha and were at least 100 m apart [9]. In each of the 45 plots we counted and identified all climbing plants rooted within the plot. No minimum size criterion was used for plant sampling. Thus, self-supported seedlings and older individuals trailing over the forest floor or climbing onto trees were counted. We included only independently growing stems not connected aboveground to other plants (apparent genets; [7]). Values of climbing plant density were reported in [9]. The present study focused on six of the seven more abundant climbers across the forest therein reported, which accounted for 91% of all climbing plant individuals [9]. The study species were *Boquila trifoliolata* (Lardizabalaceae), *Cissus striata* (Vitaceae), *Hydrangea serratifolia* (Hydrangeaceae), *Luzuriaga polyphylla* (Alstroemeriaceae), *L. radicans*, and *Mitraria coccinea* (Gesneriaceae). All of these species are woody vines (lianas). We first calculated the dominance of climbing plant species in each of the three light environments as: (relative frequency+relative density)/2 (see [15]). Accordingly, dominance values could range between 0 (least dominant species) and 100 (most dominant species). Relative frequency of species ***i*** = 100×(frequency of species ***i***)/Σ (frequencies of all species), where frequency of species ***i*** = number of plots where it occurred/15. Relative density of species ***i*** = 100×(density of species ***i***)/Σ (densities of all species), where density of species ***i*** = total number of plants of species ***i***/15×25 m^2^. In order to calculate the overall dominance across the entire light gradient for each species, and given the distribution of understory light environments in this site (it is strongly skewed towards low light, [2]), dominance values were weighed by 60%, 30%, and 10% for mature forest, secondary forest and canopy gaps, respectively, and then averaged. Thus, if species ***i*** had a dominance of 20 in mature forest, 40 in secondary forest, and 50 in canopy gaps, the overall dominance was: [20×0.6+40×0.3+50×0.1]. This overall dominance was the dependent variable to be explained by plant traits in the analysis.

For each of the six study species, we measured *in situ* four ecophysiological traits (leaf size, specific leaf area, photosynthetic rate, and dark respiration) in the two extreme light environments: mature forest and canopy gaps (N = 12 individuals per species at each light environment). To avoid spatial pseudoreplication, measured plants were located a number of meters apart, in several different plots, within a 30 ha area. Leaf size (cm^2^) was determined using digital pictures that were later analysed with Sigma-Scan Pro5 (SPSS Inc, Chicago, IL, USA). Leaves were then oven-dried at 70°C for 48 h and weighed to calculate specific leaf area (SLA, cm^2^ g^−1^). Area-based photosynthetic capacity (A_max_) and dark respiration (R_d_) rates were estimated using a CIRAS II portable infrared gas analyser and leaf chamber (PP Systems, Hitchin, UK). A_max_ was measured at PAR (Photosynthetically Active Radiation) 1000 and 700 µmol m^−2^ s^−1^ in canopy gaps and mature forest, respectively (light saturation level). R_d_ was measured at PAR 0 µmol m^−2^ s^−1^. All plant traits were measured during mid-growing season (December) on two fully expanded leaves per plant, and the average of these two measurements was used as the individual value. The secondary forest data was included in the calculation of the overall dominance to get a realistic account of plant distribution and abundance across the forest, but we did not perform physiological and morphological measurements on plants from this light environment. We rather focused on the phenotypic variation between the two “extreme” light environments, assuming that intermediate values of ecophysiological traits would be found in the secondary forest and thus would be included in the mature forest-to-canopy gap variation.

To determine the relationship between overall dominance of climbing plant species and ecophysiological traits, we conducted two multiple regression analyses. The first analysis included morphological traits and we considered four predictors: mean values of leaf size and SLA in mature forest and their phenotypic change (%) between mature forest and canopy gaps. We chose this measure of phenotypic flexibility instead of the mean values found in canopy gaps because it is more representative of the ecological scenario where plants in the prevalent closed-canopy forest should adapt to environmental changes in treefall canopy gaps (or human-made forest clearings). Likewise, the second analysis included gas exchange traits and we considered the following four predictors: mean A_max_ and R_d_ in mature forest and their phenotypic change (%) between mature forest and canopy gaps. We made separated analysis for morphological and physiological traits because there is evidence that they may play distinctive roles in plant adaptation to variation in light availability [11], [25]. Because the sample size was inevitably small (n = six species) there is a chance that the models could be overfitted because of the predictors/cases ratio (see [26]). Therefore, in the case of significant associations arising from the multiple regression analysis, we checked that the significant results were true using a different approach. Thus, for each individual plant measured in the sun (canopy gaps), we calculated the relative change in ecophysiological traits with respect to the mean value in the shade (mature forest) observed for the species. We then carried out a correlation analysis (Pearson's product-moment correlation) between these individual values and species dominance, thus evaluating whether plants from more dominant species showed reduced or increased change in ecophysiological traits from shade to sun environments. All analyses were done using Statistica (6.0, Statsoft).

## Results

**Figure 1:**
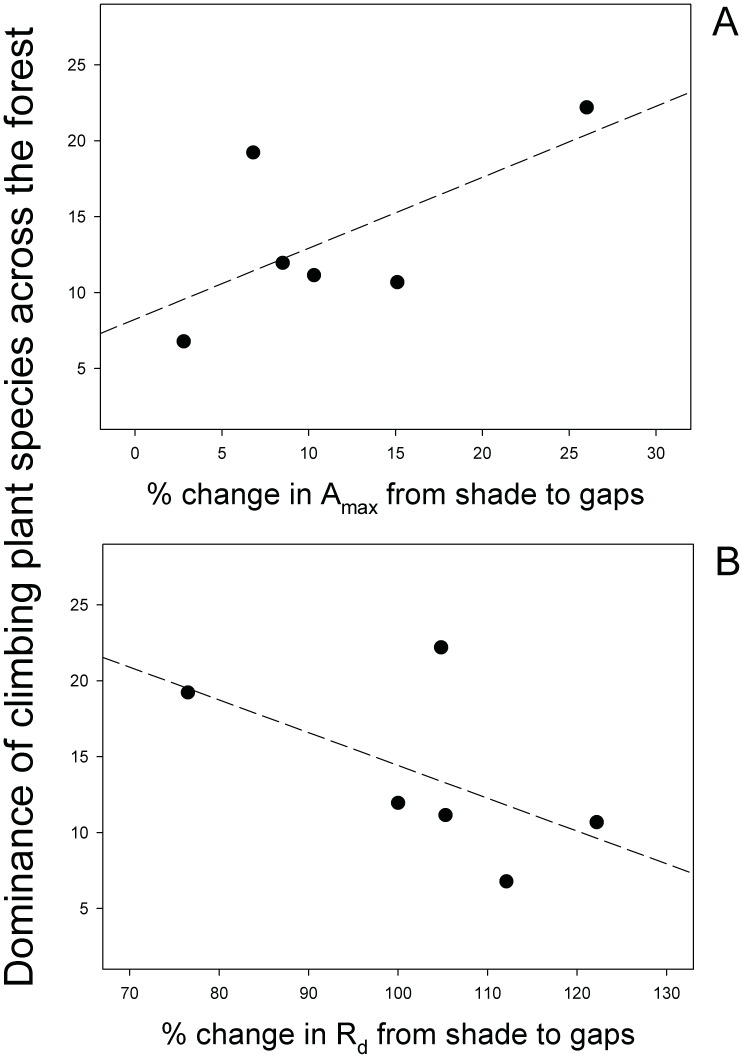
The relationship between climbing plant dominance across the light gradient in the forest and the change in ecophysiological traits from the mature forest to canopy gaps. **A**) Photosynthetic rate, A_max_; **B**) dark respiration rate, R_d_. Measurements were conducted in field plants. Each dot corresponds to a single species (n = 12 plants per species).

Dominance values and ecophysiological traits showed noticeable variation across species (Table 1). The morphological traits included in the regression analysis (leaf size and specific leaf area) were not significantly associated with climbing plant dominance across the forest (Table 2). The variation in gas-exchange traits between contrasting light environments (mature forest and canopy gaps) seemingly explained dominance of climbing plant species in this forest (Table 2). Specifically, a greater increase in photosynthetic rate and a reduced increase in dark respiration rate when canopy openings occur were related to the success of climbing plant species (Table 2, Fig. 1). Both relationships held after correlation analyses were applied (n = 72 in each case). Thus, the correlation coefficients (r) for photosynthetic and dark respiration rates were 0.23 and −0.27, respectively, and both associations were significant (P≤0.05).

**Table 1:** Dominance of climbing plant species across the light gradient and mean values of ecophysiological traits in a temperate rainforest.

		leaf size (cm^2^)		SLA (cm^2^ g^−1^)		A_max_ (µmol C m^−2^ s^−1^)		R_d_ (µmol C m^−2^ s^−1^)	
Species	Dom	for	gap	for	gap	for	gap	for	gap
*Boquila trifoliolata*	10.7	12.9±1.9	14.4±1.8	315±20	162±4	6.10±0.34	7.02±0.24	0.27±0.02	0.60±0.05
*Cissus striata*	6.8	12.9±1.7	12.6±1.3	436±41	129±14	9.27±0.99	9.53±0.79	0.33±0.02	0.70±0.04
*Hydrangea serratifolia*	22.2	20.9±2.2	33.2±4.7	212±11	86±5	5.00±0.29	6.30±0.33	0.21±0.03	0.43±0.03
*Luzuriaga polyphylla*	11.2	14.7±1.3	15.2±2.2	238±18	229±18	5.74±0.58	5.15±0.61	0.19±0.02	0.39±0.03
*Luzuriaga radicans*	19.2	15.2±2.3	20.5±3.0	195±20	258±15	4.42±0.24	4.72±0.28	0.17±0.02	0.30±0.02
*Mitraria coccinea*	12.0	7.4±1.1	6.4±0.5	260±39	216±22	4.24±0.58	4.60±0.48	0.15±±0.02	0.30±0.02
CV (%)	42.6	31.2	53.5	32.2	36.5	31.9	30.2	30.8	36.1

Overall dominance (**Dom**, average of relative frequency and relative density) of climbing plant species across the light gradient and mean values (± SE) of ecophysiological traits in mature forest (**for**) and canopy gaps (**gap**). The coefficient of variation (CV) among species for all variables is included (CV = standard deviation/mean, expressed in percentage).

**Table 2:** Relationship between climbing plant dominance and ecophysiological traits in a temperate rainforest.

Morphological traits	Beta±SE	*P*-value
Leaf size –for	0.024±0.137	0.891
Leaf size –cha	0.512±0.443	0.454
Specific leaf area –for	−0.595±0.538	0.468
Specific leaf area –cha	0.204±0.445	0.726
**Gas-exchange traits**		
Photosynthetic rate –for	−0.201±0.096	0.283
Photosynthetic rate –cha	0.845±0.038	0.029
Dark respiration rate –for	0.369±0.091	0.154
Dark respiration rate –cha	−0.899±0.038	0.027

Standardized regression coefficients (betas) of multiple regression analyses of climbing plant dominance across the light gradient against ecophysiological traits (**–for**: mean value of the trait in the closed-canopy mature forest; **–cha**: percentage of change in the trait between the mature forest and treefall canopy gaps). **Morphological traits** – Full model R^2^ = 0.963; F_4,1_ = 33.15; P<129. **Gas-exchange traits** – Full model R^2^ = 0.997; F_4,1_ = 387.59; P<0.038.

## Discussion

The mean values of the studied ecophysiological traits in the shaded mature forest were not good predictors of climbing plant dominance, despite the fact that this light environment is the predominant successional stage in the study site, and that it was accounted for in the weighted calculation of overall dominance across this temperate rainforest. Rather, the dominance of the study species, which have been described as shade-tolerant species [15], was explained –at least partly– by a higher and lower increase in photosynthetic rate and dark respiration, respectively, when exposed to enhanced light availability in treefall canopy gaps. Thus, dominant climbers show an optimal strategy of maximizing exploitation of resource availability but minimizing metabolic costs. This pattern is consistent with the “carbon gain hypothesis”, which considers that plant adaptation to light heterogeneity occurs via the maximization of light capture and use in photosynthesis together with the minimization of respiration costs for maintenance [11], [27]. It remains to be elucidated whether the reported patterns reflect phenotypic plasticity or genetic differentiation in ecophysiological traits between light environments. Plasticity in ecophysiological traits has been related to the ecological breadth of forest ferns [28] and shrubs [29], but this issue has not been addressed for climbing plants. If this is verified, climbers and self-supporting species would share functional strategies to successfully cope with light heterogeneity, despite the intrinsic differences between these growth forms [30]. It has been earlier shown that climbing plants exhibit life history trade-offs along forest light environments similar to those of trees [31], and that the relationship between photosynthetic rate and dark respiration is comparable among lianas and trees [32] (but see [10]). Because earlier work has suggested possible differences in the ecology of climbing plants in tropical and temperate rainforests (see below and [9]), further research should address the same topic in other rainforests to assess the generality of the associations reported here from a rather limited sample size.

It has been reported that woody vines are increasing in dominance, relative to trees, in both tropical [20], [21], [23] and temperate forests [22] (but see [19]). This pattern has been related to climate change [18], [33], one of the global change drivers [34], but more empirical evidence is needed. Schnitzer [7] reported that the abundance of woody vines in tropical forests is correlated negatively with precipitation and positively with seasonality. He further proposed that this pattern may be explained by the greater efficiency in water uptake and transport of woody climbers as compared to trees [7]. Our study forest is a wet, cold forest [24], where light availability is the major ecological factor affecting distribution and abundance of trees [2], [35], [36] but not of woody vines [9], [14]. In this temperate rainforest, where the potential evapotranspiration is very low, water availability is not a limiting factor and therefore water use features are unlikely to determine plant distribution and abundance. From an applied perspective, the results of the present study suggest that the dominant climbers in the southern temperate rainforest could be able to cope with another global change driver, land-use change, if forest clearings occur due to human activities.
